# The four principles: Can they be measured and do they predict ethical decision making?

**DOI:** 10.1186/1472-6939-13-10

**Published:** 2012-05-20

**Authors:** Katie Page

**Affiliations:** 1 School of Public Health, Institute of Health and Biomedical Innovation, Queensland University of Technology, Australia

**Keywords:** Ethical principles, Hierarchies, Medical ethics, Analytic hierarchy process

## Abstract

**Background:**

The four principles of Beauchamp and Childress - autonomy, non-maleficence, beneficence and justice - have been extremely influential in the field of medical ethics, and are fundamental for understanding the current approach to ethical assessment in health care. This study tests whether these principles can be quantitatively measured on an individual level, and then subsequently if they are used in the decision making process when individuals are faced with ethical dilemmas.

**Methods:**

The Analytic Hierarchy Process was used as a tool for the measurement of the principles. Four scenarios, which involved conflicts between the medical ethical principles, were presented to participants who then made judgments about the ethicality of the action in the scenario, and their intentions to act in the same manner if they were in the situation.

**Results:**

Individual preferences for these medical ethical principles can be measured using the Analytic Hierarchy Process. This technique provides a useful tool in which to highlight individual medical ethical values. On average, individuals have a significant preference for non-maleficence over the other principles, however, and perhaps counter-intuitively, this preference does not seem to relate to applied ethical judgements in specific ethical dilemmas.

**Conclusions:**

People state they value these medical ethical principles but they do not actually seem to use them directly in the decision making process. The reasons for this are explained through the lack of a behavioural model to account for the relevant situational factors not captured by the principles. The limitations of the principles in predicting ethical decision making are discussed.

## Background

“But I think the four principles should also be thought of as four moral nucleotides that constitute the moral DNA - capable alone or in combination, of explaining and justifying all the substantive and moral norms of health care ethics and I suspect of ethics in general” [1], p.308

There is no denying the impact and importance of the medical ethical principles in medical ethics, or the high esteem in which they are held - as the quote from Gillon [1] illustrates. Since their introduction by Beauchamp and Childress [2] they have been the dominant approach to the teaching and evaluation of medical ethical dilemmas in health care. Whilst they have received some criticism, predominantly from the casuists (the other main method adopted in bioethics) [3,4] they are still widely used and discussed, even despite their limitations, both in practice and in the academic literature. However, given their dominance as an approach to medical ethics surprisingly little work has been conducted on the empirical importance and merit of the principles for individuals. Specifically, empirically establishing the worth of the principles as a descriptive and explanatory framework has received minimal academic attention. This may be because of the difficulty associated with the quantification of the principles and/or the focus on more practical and case specific goals. Whatever the case, the scope for further investigation is large, and given the importance of medical ethics and understanding ethical outcomes in the medical sphere, it is also of considerable academic and clinical importance. This study develops a measure of the medical ethical principles using the Analytic Hierarchy Process (AHP) [5] as a methodological tool. It then tests whether the principles are predictive of ethical judgements.

### Medical ethical principles

Research on the importance of medical ethical principles is generally one of three types. The first type, where most of the research has been focused, concerns the moral development and orientation of medical students throughout their medical training [6-9]. However, this body of literature is mainly focused on the developmental nature of morality rather than the specific principles valued, or their role in personal ethical decision making.

The second type of research has examined differences between groups of students with respect to the ethical principles they value [10,11] and compares whether the same principles are important for different professions. These findings have shown that females value autonomy more than males and that medical students are generally more beneficent than lawyers.

The third kind of research investigates medical students’ evaluations of certain ethical principles [8,12]. The study by Herbert [12] had students read several ethical scenarios and identify as many ethical issues as they could in each scenario. The responses were evaluated *post hoc* against a “gold standard” marking scheme, where issues were classified as reflecting one of the three principles of autonomy, justice and beneficence. Results indicate that students were able to identify ethical issues in the cases consistent with the principles, however, the responses were often limited in range and tended to be of the same type. Price, Price, Williams and Hoffenberg [8] examined changes in medical students’ attitudes as they progressed through their medical course, where attitudes were defined by the assessment of the ethical principles.

Westin and Nilstun [13] discuss the principles and whether a certain type of ethical conflict (patient with ischemic heart disease) could be resolved by using “normative” reasoning with the ethical principles. This study was useful in identifying the conflicting nature of the principles for different stakeholders and the difficulties encountered with applying them in practice to a specific case, but it did not take an empirical approach to identifying the importance of the principles and their application. Moreover, it has been criticised for the manner in which the principles were applied or interpreted (principles examined in isolation) in the given case [14].

Landau and Osmo [15] investigated the hierarchies of ethical principles among Israeli social workers. The ethical principle of “Protection of life” was the most important principle guiding the social workers’ decision making with 45% of respondents rating this principle as the most important. However, there was little consensus among the rankings of the ethical principles by the social workers and there was no consistent pattern of application across cases. Professional and personal hierarchies were not significantly different from each other. Social workers responded mainly to the case information and changed the importance of their ethical principles based on the situational information available in each case.

Twelve ethical principles were used in the Landau and Osmo [15] study including “Autonomy & freedom”, “Truthfulness & full disclosure”, “Least harm” and “Public good”. The principles overlap considerably with the four principles but had a more specific social work focus. The 12 principles are also not conceptually distinct. The present study focuses only on six principles with an effort to prevent any conceptual overlap between principles. From a methodological point of view, the Landau and Osmo [15] study was based only on the ranking of principles and simple Likert scales. The present study proposes an additional way to assess the importance of principles by computing the relative weight of principles for each individual. This has the advantage of being able to assess the importance of the principles when two very important principles conflict, which was not the case in the Landau and Osmo [15] study.

Because of the novelty of this approach and the introduction of a new measure, no specific predictions can very clearly be extrapolated from past findings or empirical research. However, there is considerable theoretical discourse concerning the importance of the principles in medical ethics. Several arguments and pieces of evidence exist that may point to the possible empirical results.

First, the debate on medical principles can indicate which medical principles are the most important. In the Hippocratic Oath the principle of primary importance is *Primum non nocere*, above all do no harm. This principle has a long history of being very important in health care contexts. The Hippocratic injunction to do no harm has been an axiom central to the education of medical and graduate students [16].

In more recent times, the primary importance of this principle has been contested. In a reflection and discussion of the principles and their merits, Gillon [17] argues for the principle of autonomy to ‘trump the rest’. He provides compelling reasons why respect for autonomy should be of primary importance in medical ethics and applied ethics in general. Different people argue for different hierarchies and yet others argue for no hierarchy at all [18], just the application of all the relevant principles to a case on a more relativistic basis. Prediction from the theoretical literature is therefore not straightforward.

In summary, this study examines the medical ethical principles and uses the AHP as a methodological tool to derive individual weightings for ethical principles. There are two aims of this study. First, to develop and evaluate a measure of the four principles from medical ethics, and second, to determine whether individuals’ rankings of these principles are used in decision-making in ethical scenarios where these principles conflict.

### Analytic hierarchy process: an overview

The measure of the medical ethical principles developed here uses pairwise comparisons to elicit the weightings for the principles. This methodology is part of the AHP. The AHP is a multi-criteria decision making tool originally developed by Saaty [5] that has been widely applied to many areas in the field of decision making [19] including resource allocation [20], business performance evaluation [21], project selection [22], and auditing [23].

In the AHP, a judgement or a comparison is the numerical representation of a relationship between two elements that share a common parent. In this study there is only one parent (ethical principles) and a judgement consists of a rating of the relative importance of one principle over another. Through trade-offs the technique enables the explication of the advantages and disadvantages of options under circumstances of risk and uncertainty.

The AHP is used in this study as a pragmatic tool to assess the relative preferences that individuals have for the principles. The technique of weight computation for the principles can be considered an alternative way to assess the importance of the principles in the individual decision making process. Prior research has tended to only measure the importance of principles either in scenarios, in isolation (one principle at a time), or with post-hoc matching of responses to set criteria. The AHP methodology is a novel approach in this area.

It should be noted that no behavioural hypothesis about the way people cognitively use the principles is made in order to use the AHP. The numerical results must therefore be seen as an approximation and, to some extent, still qualitative, in spite of their quantitative nature.

## Methods

### Participants

Participants were 94 first year psychology students from the University of Queensland, Australia. Participation in this study formed part of their course requirements. There were 65 females (69%) and 29 males. The age of the participants ranged from 17 to 58 years with an average age of 21.5 (SD = 7.01) years. Eighty-two percent of participants were Australian and the other 18% were from predominantly Asian backgrounds. This study was cleared in accordance with the ethical review processes of the University of Queensland and within the guidelines of the National Statement on Ethical Conduct in Human Research, clearance number 03-PSYCH-P-02.

### Measure of the medical ethical principles

The measure of medical ethical principles that uses the AHP methodology was designed to measure the importance of the medical ethical principles in a general and global sense, that is, in a context without specific situational information or cues. In the past, given the complicated nature of the principles and the importance of all of the principles, it has generally been the case that they have been discussed and debated in situations where the principles come into conflict within a specific case scenario. The scenario is then assessed from a principles perspective as the extent to which the principles bear on the case. This approach is understandable and often informative but in this study the interest is in knowing whether people hold more general preferences for the principles that supersede specific case information. That is, do people weight some of the principles as more important than others irrespective of the situation at hand?

The four standard principles proposed by Beauchamp and Childress [2] were used in the new measure, as well as two other principles; confidentiality and truth-telling, which are within the Beauchamp and Childress [2] framework embedded within the principle of autonomy. The concepts are often discussed separately in medical ethical discourse, and they exist as separate entities in the medical code of ethics from the American Medical Association. Moreover, previous empirical research has also separated these principles [10,15]. Therefore, the new measure was developed to assess the importance of six medical ethical principles; non-maleficence, beneficence, autonomy, justice, confidentiality and truth-telling.

For each principle statements were developed that defined the principle in a general sense without reference to characteristics of a specific situation. This provided participants with precise knowledge of the principles necessary for making an informed and accurate judgement. There were two statements for each principle, each expressing the concept in a slightly different way with essentially the same meaning - for example: Beneficence - An obligation to convey benefits and to help others to further their legitimate interests (i.e. “One has a moral obligation to help other people”). For each principle there was also two examples of the principle to demonstrate the meaning in a broad context, one for medicine and the other for business. The measure is a pair-wise ranking task that forces participants to choose between the ethical principles (in the form of generalised statements) when they conflict. All possible pairs are formed from the six principles, making a total of 15 pairs of statements. In this way, the measure allows the assessment of the relative importance of the principles. The measure is analysed using the AHP [5] which yields relative weights for the six principles. In addition to this new measure, participants were also asked to rank order the principles from most to least important.

### The scenarios

There were four scenarios used in this study all containing ethical issues framed in a medical context and involving medical ethical principles. The first was an IVF scenario dealing with issues of ownership, autonomy, and privacy. Two scenarios (hereafter referred to as Confidentiality and End of Life) were from a questionnaire on medical ethics [8]. The Confidentiality scenario primarily concerns issues of privacy and trust (Dr Heron has a right to his confidentiality) weighed against the principle of non-maleficence (the possibility of future harm to potential patients). In contrast, the End of Life scenario concerns patient autonomy and the right of a patient to choose to end their own life. The ethical conflict in this case arises because of the conflict between autonomy and professional duty and non-maleficence. The fourth scenario is a commonly cited and discussed case in the field of medical ethics and involves the process of a blood transfusion for a child of Jehovah’s Witnesses [24]. This case involves the principles of beneficence (helping the child’s interests) versus patient autonomy or the parents’ right to decide for their child. Together these four scenarios were thought to provide a good basis for, and be representative of, the salient issues in medical ethics. All four scenarios can be seen in Additional file 1. At the end of each scenario participants were asked two questions, the first about the ethicality of the action (1) How ethical is this action? (rated on a seven point Likert scale from very unethical to very ethical), and the second concerning their intentions to act in that way if they were in the same situation, (2) I would act in the same way (rated on a seven point Likert scale from strongly disagree to strongly agree).

### Demographic questionnaire

The demographic questionnaire consisted of several items which asked questions about age, gender, religious commitment, and ethical training. Responses were given in either a generated written format or by circling or ticking an appropriate scale response option. These items were included in order to control for the effects of some possible explanatory variables (age and gender) and/or to investigate gender differences where appropriate.

### Procedure

Questionnaire booklets were compiled containing the measures outlined above and these were administered to participants in a classroom environment by an experimenter. The ordering of the materials remained consistent across participants with the demographic measure first, followed by the scenarios, and finally a measure of medical ethical principles was presented last. Participants were given a maximum of one hour for the completion of the task.

## Results

### The pairwise matrices

From the list of 15 pairs of statements, a reciprocal matrix was constructed for each person from the preferences that participants indicated (see Table 1). For example, if the participant rated principle A (“Autonomy”) as preferable to principle B (“Beneficence”) by a strength of “5” then the upper section of the matrix would be a 5 and the corresponding lower cell would be 1/5. This would be repeated for all cells until a full reciprocal matrix was obtained. Each column and each row represent one of the six medical principles so the resulting matrix is a 6×6 reciprocal asymmetric matrix where each number represents the participants preference for one principle over another and by a given magnitude on a scale from 1-9.

**Table 1:** General representation of the matrix form of the medical ethical principles using Saaty’s (1980) pairwise task, and example matrix and weights for Person A

	**2**^***nd***^**position (*non-preferred*)**								**Person A**						
		**NM**	**J**	**A**	**B**	**TT**	**C**		**NM**	**J**	**A**	**B**	**TT**	**C**	**Weights**
	**NM**	–	*W*1	*W*2	*W*3	*W*4	*W*5		–	$\frac{1}{3}$	1	1	5	2	0.17
	**J**	$\frac{1}{W1}$	–	*W*6	*W*7	*W*8	*W*9		3	–	$\frac{1}{3}$	1	5	2	0.20
**1**^**st **^**position (*preferred*)**	**A**	$\frac{1}{W2}$	$\frac{1}{W6}$	–	*W*10	*W*11	*W*12		1	3	–	7	1	1	0.27
	**B**	$\frac{1}{W3}$	$\frac{1}{W7}$	$\frac{1}{W10}$	–	*W*13	*W*14		1	1	$\frac{1}{7}$	–	5	$\frac{1}{5}$	0.11
	**TT**	$\frac{1}{W4}$	$\frac{1}{W8}$	$\frac{1}{W11}$	$\frac{1}{W13}$	–	*W*15		$\frac{1}{5}$	$\frac{1}{5}$	1	$\frac{1}{5}$	–	$\frac{1}{5}$	0.06
	**C**	$\frac{1}{W5}$	$\frac{1}{W9}$	$\frac{1}{W12}$	$\frac{1}{W14}$	$\frac{1}{W15}$	–		$\frac{1}{2}$	$\frac{1}{2}$	1	5	5	–	0.19

NM = Non-maleficence, J = Justice, A = Autonomy, B = Beneficence, TT = Truth Telling, C = Confidentiality.

An SPSS syntax file was generated for the purpose of calculating the matrices, the corresponding preference weights for each person and their consistency index. These calculations were derived from the formulas presented by Saaty [5]. For example, take a person A, whose answers correspond to the matrix presented in Table 1. The weights shown in the last column are computed from the 6 × 6 matrix using the AHP. In this example, A prefers *autonomy* most, making it his or her strongest principle, with truth telling their least important principle.

### Overall weightings of the ethical principles

Table 2 shows the weights for the six ethical principles for the entire sample. The weights seem to indicate that five of the principles are equally important because of the roughly equal weightings. Moreover, there is clearly a preference for non-maleficence over the other five principles. This implies that when in conflict there is a strong preference for causing no harm as an *a priori* principle.

**Table 2:** Mean weightings and standard deviations for the six medical ethical principles and self-report rankings of the most and least important principles

**Medical Principle**		**Most Important**		**Least Important**	
	$\overset{̅}{\text{X}}\text{(SD)}$	**N**	**%**	**N**	**%**
**Non-maleficence**	.25 (.12)	54	57.4	9	9.5
**Justice**	.16 (.09)	47	50.0	10	10.6
**Autonomy**	.16 (.10)	21	22.3	28	29.8
**Beneficence**	.15 (.10)	23	24.4	33	35.1
**Truth Telling**	.12 (.10)	16	17.0	46	48.9
**Confidentiality**	.16 (.11)	15	15.9	58	51.1

N = 92.

Participants were also asked to provide their own rankings of the six principles on a scale from least important to most important. The percentages of people who ranked the principles in one of the first two positions of importance and in one of the last two positions are also shown in Table 2.

These self report rankings are relatively consistent with the rankings from the AHP. Approximately 57% of participants rank the principle of non-maleficence as one of the two most important principles and this dominance is reflected by the high weighting in the AHP task. The principle of justice is also rated as very important by 50% of the sample however, this principle, as measured by the AHP method, is less important than the self report measure would indicate. Both truth-telling and confidentiality are the least important principles using the self rankings. Again, this is partially consistent with the AHP rankings.

A series of paired sample t-tests with bonferroni corrections were conducted to test the differences between the weightings. The results of this analysis can be seen in Table 3. There are significant differences between non-maleficence and all of the other principles. There are also significant differences between autonomy and truth-telling, justice and truth-telling and confidentiality and truth-telling. Therefore, non-maleficence is the most important principle and truth-telling the least important principle.

**Table 3:** Results from paired sample t-tests for the medical ethical principles

**Pair**	$\overset{̅}{X}$**diff**	**SD**	**t**
Non-maleficence vs Justice	.09	.18	4.9^2∗∗∗^
Non-maleficence vs Autonomy	.09	.16	5.4^6∗∗∗^
Non-maleficence vs Beneficence	.11	.17	6.1^7∗∗∗^
Non-maleficence vs Confidentiality	.10	.20	4.7^5∗∗∗^
Non-maleficence vs Truth Telling	.13	.18	7.0^5∗∗∗^
Autonomy vs Justice	.00	.15	-.07
Autonomy vs Beneficence	.01	.16	.83
Autonomy vs Confidentiality	.00	.13	.36
Autonomy vs Truth Telling	.04	.15	2.4^1∗^
Justice vs Beneficence	.01	.15	.96
Justice vs Confidentiality	.01	.17	.34
Justice vs Truth Telling	.04	.14	2.5^2∗^
Beneficence vs Confidentiality	-.01	.17	-.50
Beneficence vs Truth Telling	.02	.14	1.52
Confidentiality vs Truth Telling	.03	.16	1.8^8 + ^

^+^*p *< .1, ∗ *p *< .05, ∗∗ *p *< .01, ∗∗∗ *p *< .001.

### Gender differences and individual heterogeneity

A one-way ANOVA was conducted to test if males and females differed in their AHP weightings of the ethical principles. Results revealed that the only difference between the sexes was for the truth-telling principle such that females ( $\overset{̅}{X}=.14$) weighted the principle as significantly more important than males ( $\overset{̅}{X}=.09$), *t*(90) = −1.93,*p *< .05. There was also a significant correlation between years of university education and the ranking of the autonomy principle. People who have spent more years at university rate the principles of autonomy as more important, *r *= .21,*p *< .05.

While overall the most important principle is non-maleficence, there is considerable heterogeneity between people. The majority of the sample has high weightings for non-maleficence over the other principles but there are sub-groups of people whose pattern of weightings are quite different. Table 4 shows the weightings for three people. Their profiles are quite different. Person B represents the more typical member of the sample with a strong preference for non-maleficence over the other principles. Person A, by contrast, has a more balanced profile with the strongest preference for autonomy but also a strong preference for justice. Person C holds an altogether different uncommon profile with a very strong preference for confidentiality and a second less strong preference for truth-telling.

**Table 4:** Comparison of weights for Person A, B and C derived from their respective matrices

**Principle**	**Person A**	**Person B**	**Person C**
Non-maleficence	.17	.48	.07
Justice	.20	.15	.02
Autonomy	.27	.07	.14
Beneficence	.11	.03	.04
Truth Telling	.06	.24	.25
Confidentiality	.19	.02	.47

Person A, CR = .35 Person B; CR = .40; Person C, CR = .32.

The AHP methodology also yields a consistency ratio (CR), which is an indication of how consistent people are in their judgements (0 being perfectly consistent and 1 being totally random). Saaty [5] recommends a threshold of .10 for this index. In this task people were much more inconsistent with an average CR of around .30 ( $\overset{̅}{X}=.32,\mathrm{SD}=.26$). However, when the most inconsistent people were removed from the sample and the average weights recalculated, the order and magnitude of the weights remained unchanged.

Moreover, because people are inconsistent does not imply that they are not doing the task properly. It may be the case that there is more than one dimension on which they are basing their judgements of the principles. The AHP methodology is not sensitive to this distinction because it uses only the first eigenvector and eignevalue. In general, these results show that considerable heterogeneity can exist between people in their preferences for the principles.

### Predicting ethical judgments and intentions

The correlations between judgements for the four medical ethical scenarios, and the medical ethical principles are shown in Table 5. There are no significant correlations between the weightings of the principles and the participants’ ethical judgements. These relationships were also explored for participants’ ethical intentions and the same results were obtained.

**Table 5:** Correlations between ethical judgements and the medical ethical principles

	**Scenario**			
	**IVF**	**Blood**	**Confidentiality**	**End of Life**
**Non-maleficence**	.11	.11	.01	-.02
**Justice**	.01	-.03	-.07	.01
**Autonomy**	-.04	-.01	-.01	.12
**Beneficence**	.01	.06	.12	-.06
**Truth Telling**	-.17	.00	-.14	-.05
**Confidentiality**	.00	-.15	.07	.00

The absence of any relationship between preferences for medical ethical principles and scenario judgements precludes the possibility of the principles predicting judgements and intentions. This lack of predictiveness for the medical ethical principles may be due in part to the limited variance in the weightings. Also, the calculation of the weights using the method described by Saaty [5] results in small dependent weights with limited variance. This can be problematic in regression equations.

## Discussion

Using the AHP to measure the relative importance of the different medical ethical principles for individuals, the most important principle is, without ambiguity, “Non maleficence”. The weight of this principle is twice as large as any of the other principles. The other principles (“Autonomy”, “Justice”, and “Truth telling”) have roughly similar weight, with “Truth telling” being the least important principle. These results are consistent with those of Landau and Osmo [15]. In their study “protection of life” was the most important principle and this seems to overlap conceptually with the principle of non-maleficence.

Interestingly, the weights elicited with abstract questions about the principles (independently from contextual information) have no predictive power to explain the participants’ choices in specific scenarios. This is also in concurrence with Landua and Osmo [15] findings, which suggest that the application of principles in scenarios is not consistent because the principles are not related to ethical judgments. Therefore, situational information seems to be of greater importance, which is in support of more casuistical claims. There are a number of reasons for this lack of prediction. First, the scenarios used to elicit judgements in this study may not have been sufficiently clear cut in terms of the conflict between principles. Scenarios with clearer conflicts may result in better predictions or significant relationships. This explanation seems unlikely though given that at least one of these cases is used extensively within the bioethics literature to demonstrate clear conflict between principles.

Second, it could be that in terms of predicting ethical outcomes the principles may only be useful when evaluated (rated) in the context of a specific scenario. Perhaps situational information, in all its complexity, is such that it “re-weights” the principles, and general weightings are rendered somewhat arbitrary in the face of new specific case-based information, as seemed to be the case in the [15] study. When participants were faced with these cases they may have used the situational information to derive the importance of the principles (or approximation of) in a more casuistical reasoning manner. This explanation does assume that individuals are relying on principles in some form to guide their judgments. Whether the principles they use are those of of Beauchamp and Childress is debatable.

Third, it could be that these principles are related to other more specific constructs that help shape decision making. For example, these principles may be used in the formation of moral norms (A moral norm measures the personal obligation felt toward adopting a behaviour [25]). There is some evidence to suggest that these ethical principles are linked to moral norms [26]. Specifically, Blondeau, Godin, Gagnea and Martineau [26] found that the principle of beneficence was linked to moral norms in the case of organ donation, and moral norms is a strong predictor of intention to adopt certain behaviors [27,28]. This means that more general variables that are of an ethical nature can play an important role in structuring the motivation to adopt specific behaviors. Therefore, aiming to measure these principles is an integral part of the process of determining which principles are important and under what circumstances they are adopted.

Overall, and more holistically speaking, these results pose some questions for the importance and use of the principles in an empirical and applied sense. Their worth in terms of conceptualising the moral issues in a scenario seems obvious but if they are not actually used, or able to be used, in decision making by clinicians then it raises questions about their overall utility and applicability (at least in their current guise). There is an ongoing theoretical debate in the bioethics literature concerning the relative merits of the two main methodological approaches: principalism and casuistry. This study highlights this ongoing tension and also reinforces the need to find a middle ground in terms of progressing forward. One approach to this progression is to adapt principalism, in the sense proposed by DeMarco [29], who introduces the notion of a mutuality principle as a way of giving principalism a greater moral coherence and future perspective. The other approach is to adapt casuistry in order to develop more usable/case-driven principles [30]. It remains unclear as to the best way forward, if indeed there is a “best” way.

It seems to me that both of these attempts at finding a middle ground are likely to suffer as long as the focus remains on finding a normative/coherent solution. I suggest that a much more useful strategy is to empirically test the two approaches and to derive a behavioural model that can more accurately describe and predict both how and when these sorts of principles are likely to be used and what other principles may be important.

## Conclusions

Overall, this study shows that people state they value these ethical principles but they do not actually use them directly in the decision making process. It is possible that people do not base their decisions in ethical situations on abstract ethical principles, and that they only respond to very unique situational information. However, I think it is most likely that the absence of predictive power of the principles in this study is due to the absence of a behavioural model explaining how individuals cognitively use these principles in their decision making. As stated initially, the AHP makes it possible to gain a qualitative approximation of the importance of these principles for individuals. A full understanding of their role in medical decision making would require a behavioural model. Future work could look at how they are actually integrated into the decision making process, and whether these or other principles are used. In general, empirical studies of this nature can help to define the scope of use for the principles and determine the level at which they are applied (if at all) in the decision making process. Such work is essential to complement, inform, and test the normative claims of principalism.

## Competing interests

The author declares that she has no competing interests.

## Pre-publication history

The pre-publication history for this paper can be accessed here:

http://www.biomedcentral.com/1472-6939/13/10/prepub

## Supplementary Material

Addtional file 1 Four medical ethical scenarios. Click here for file
